# Physicians’ Advertisements

**Published:** 1866-10

**Authors:** 


### Physicians' Advertisements.

(From the Boston Medical and Surgical Journal.)

The frequent abuse of the mistaken leniency which allows a physician publicly to announce or recommend himself has become so frequent among us of late as to call for some notice from us, and for stringent action on the part of our State Medical Society. Until recently the announcement of a change of residence, or of a resumption of practice, was the extent of what was considered justifiable by the laws of professional decorum, although even these exceptions have sometimes overstepped the limits of propriety by being kept too long before the public eye; but now we find newspapers of this city every day containing the advertisements of members of our body which can in no way be distinguished from those of some professional quacks. Not satisfied, moreover, with seeking notoriety by special and extra puffs in the columns of the daily journals, disgraceful exhibitions of machinery, and written promises to cure are disgracefully presented to the gaze of the passer-by in office windows, pamphlets containing accounts of wonderfully successful cases are published for public distribution, and self-laudatory circulars are issued for the medical reader.

There is another class of advertisements which is also becoming more frequent, and which, although hitherto considered at least not improper by some of the profession, is so liable to abuse in its present undefined condition, that it has become a matter worthy of grave consideration. We mean the cards of so-called specialists published in a medical journal. It is a custom which has so gradually sprung up in this city as to have become an almost recognized law among us, and we do not in the least intend to reflect upon the gentlemen who have availed themselves of its privileges. It is a custom, however, which is confined to ourselves, and which would be considered entirely unprofessional in any other part of the country. Why, then, is it allowed here? It has been urged in its favor, that it is for the public good that the profession should be made acquainted with the fact of the existence of those who have particularly studied those branches of our science which require for their mastery more time than is at the command of the general practitioner. There can be, of course, no question as to the advantage, the necessity even, of a division of labor in the study and practice of medicine. It is so well recognized in all the sciences, that we do not propose to discuss it at all. It is also desirable that the profession should know who are the most skilled in these special branches, that patients may be entrusted to those who can employ in their behalf this superiority of training. The question is, in what way shall the profession gain this information? It is evident that the mere announcement, by a published card, that Dr. So-and-so gives his specialor exclusive attention to this or that class of diseases in no way indicates his fitness or professional standing. Specialties are multiplying rapidly, and are taken up after so little preparation, in many instances, as a ready road to celebrity and wealth by the young practitioner, that the intimate knowledge of the character, education and experience of the claimant for the public confidence has become essential. It is evident that this can not be obtained from, or imparted by, the advertisement of the interested party, and that if all specialists advertised themselves, the public and general practitioners who are unacquainted with the professional reputation of the individual must, in some cases, find it as difficult to make choice of the person to whom they shall commit themselves or patients as if no such announcement were made; not to mention the possibility of selecting one whose name would never have reached their ears through the recommendations of their professional brethren. But all specialists do not advertise. There are those who look upon the ordinary announcement upon the covers of a medical journal as a self-recommendation to the public, and who believe that the specialist is no exception to the general rule that each man's success should rest wholly upon his own merits. Evidently those who think differently gain a great advantage at the start over the latter, which would not be the case if one rule regulated the conduct of all. Whether the custom be indirectly beneficial to the profession or not, there can be no doubt as to to the self-interested motive of the advertiser.

